# Relationships between diversity demographics, psychological distress, and suicidal thinking in the veterinary profession: a nationwide cross-sectional study during COVID-19

**DOI:** 10.3389/fvets.2023.1130826

**Published:** 2023-08-16

**Authors:** Kristel Scoresby, Carrie Jurney, Amanda Fackler, Christina V. Tran, William Nugent, Elizabeth Strand

**Affiliations:** ^1^College of Social Work, University of Tennessee, Knoxville, TN, United States; ^2^Not One More Vet, San Francisco, CA, United States; ^3^Remedy Veterinary Specialists, San Francisco, CA, United States; ^4^Multicultural Veterinary Medical Association, Silverdale, WA, United States; ^5^College of Veterinary Medicine, University of Arizona, Tucson, AZ, United States; ^6^Veterinary Social Work, Colleges of Veterinary Medicine and Social Work, University of Tennessee, Knoxville, TN, United States

**Keywords:** diversity, race, gender, sexual orientation, well-being, distress, suicide

## Abstract

**Purpose:**

This study aimed to determine the relationship between demographic diversity and veterinary professionals regarding their psychological distress and suicidal experiences. This study also aimed to determine what demographic factors were associated with psychological distress and suicidal experiences for veterinary professionals.

**Methods:**

This study used a cross-sectional web-based questionnaire to assess the prevalence of diversity, psychological distress, and suicidality in individuals over 18 working in the veterinary field within the United States. The study received 2,482 responses resulting in 2,208 responses that were included in the analysis. Descriptive statistics were performed to identify the categories with the highest rates of psychological distress, suicidal thoughts, and suicidal behaviors. Binomial logistic regressions were conducted to identify the strongest statistical predictors of psychological distress (Kessler-6-K6), suicidal thinking and suicide behaviors.

**Results:**

Of the 2,208 respondents included in the analysis, 888 (41%) were experiencing serious psychological distress and 381 (17.3%) had considered suicide in the past 12 months. Results of the binomial regressions indicate gender, social class, age, and disability status were the strongest predictors of psychological distress. When controlling for psychological distress, the strongest predictors of suicidal thinking were sexual orientation, marital status, and professional role.

**Implications:**

Limited research has been done to explore the relationship between demographic diversity of veterinary professionals and psychological distress, suicidal thoughts, and suicidal behaviors specifically. These results shed light on multiple demographic factors that promote and attenuate mental health, as well as the importance of asking respondents their demographic identities in veterinary medicine research. This research attempts to identify these mental health factors without collapsing categories with small sample sizes, which does cause a limitation in statistical power, yet also demonstrates how to increase inclusivity in research.

## Introduction

Veterinary professionals are at higher risk of mental health concerns and suicidality. Data on suicidality of veterinary professionals spans decades, first being mentioned in a review of causes of death of US veterinarians from 1947 to 1977, which showed white male veterinarians had a 1.7-fold risk of death by suicide compared to the general public (1). Elevated risk of death by suicide was expanded to include female veterinarians in a review of the causes of death in California veterinarians spanning from 1960 to 1992, where male and female veterinarians were shown to be at 2.5 and 5.9 times the risk of the general public to die by suicide (2). This elevated risk level is reflected in literature from several countries, including Norway, the United Kingdom, Belgium, and Australia (3–6). Literature regarding suicidal behavior and the veterinary profession has primarily focused on veterinarians. However, a 2019 study showed that veterinary technicians were also at significantly higher risk of dying by suicide when compared to the general public, with male and female technicians having a 5 and 2.3 fold risk, respectively (7).

The cause of this increased risk is multifactorial, and the literature reflects a variety of personality and occupational stressors. A variety of mental health concerns are reported in veterinarians. In a study of US veterinarians, male and female veterinarians were found to have elevated levels of serious psychological distress (6.4% and 11.0% respectively) when compared to the general public (8). The percentage of men and women in the general public that were shown to have high psychological distress were 2.4% and 3.9% (9). A study of over 5,000 US veterinarians found 50.2% had low mental well-being scores, and 58.9% had high secondary traumatic stress scores (10). Broadly, the concerns include personality factors, financial concerns, interpersonal conflict and coworker dynamics, client interactions, work-life balance concerns, exposure to euthanasia, and access to means (7, 11–14). One personality factor that warrants particular attention is neuroticism-also known as *emotional instability.* This is one of five factors in the Big Five personality traits model-the others being agreeableness, conscientiousness, extraversion, and openness (15). Neuroticism is associated with experiencing more negative emotions and being more sensitive to stress (16). It is not only associated with having more negative mental health experiences generally (17), but was also the strongest predictor of psychological distress and low wellbeing in a representative sample of United States veterinarians (18). Rates of this personality factor were also higher in veterinarians than in the general population (18), making this a particularly important part of understanding mental distress and low well-being in veterinarians. Perfectionism, which has been shown to be strongly correlated with neuroticism, is also associated with decreased resilience and enhanced experience of moral stressors in veterinary professionals (19, 20).

### Diverse demographics

Another aspect of human experience that can contribute to increased psychological distress and suicidal thoughts is an individual’s social location and identity. Given the events surrounding racism in 2020, even more pressure has been put on researchers to update and expand demographic questions on surveys for two purposes: (1) to increase research integrity by accurately describing a sample and (2) to be inclusive of the multiple social identities a research participant may be experiencing (21). Previous research has evaluated mental health in veterinary professionals through the demographic lens of age, binary gender (male or female), and income (12, 13, 18, 22, 23). However, multiple other demographics are missing in the literature. Recently, a study was conducted that identified psychological distress in veterinary professionals who identified with diverse gender and sexual orientation identities (24). Findings indicated that transgender, nonbinary, non-heterosexual cis women and non-heterosexual cis men had even higher rates of suicidal ideation and psychological distress than previously reported binary (male or female) veterinarians. To our knowledge, Witte’s study is the first published study to look at diverse gender and sexual orientation demographics.

Race and ethnicity are also important to consider. In the general population, race and ethnicity are known factors that correlate with worse physical and mental health (9, 25, 26). There are several pathways by which these outcomes occur such as structural, cultural, and individual racism (27). The profession of veterinary medicine is predominantly a white non-Hispanic population. Rates of this veterinary demographic are higher than in the general USA population. For instance, estimates in 2020 indicate 87.5% of USA veterinarians were non-Hispanic whites, compared to 61.9% of people in the general population. Among veterinary technologists rates are similar: 73% of veterinary technicians were non-Hispanic whites, compared to the general population which was 61.9% (28). Although there has been a steady increase of veterinary students with racial and ethnic diversity since the 1980’s, in 2020 the prevalence of racially diverse students in veterinary colleges was somewhere between 25%–30% (29) whereas among human medical students it was 49% (30).

Disability status should also be considered a demographic identity. Individuals who experience a disability are more likely to experience psychological distress than those without a disability (9, 31, 32). In veterinary medical education it can be a challenge to successfully attend to student learning differences due to disability status while also meeting curricular competencies, particularly the clinical year (33). Having to access these accommodations within veterinary medical education can be difficult for some students who may not want others to be aware of their unique learning needs.

Marital status is also included as a demographic identity as research often indicates that the built-in support through marriage is a protective factor for mental health and that those who are separated, divorced, never married, or widowed are at higher risk of psychological distress (34, 35). However, in the veterinary profession, cisgender female veterinarians report feeling less career support from their spouses and increased marital stress than cisgender male veterinarians (36). In a study of veterinary officers, higher scores of burnout, depression, and anxiety were reported for married veterinarians than for single veterinarians (37).

### COVID-19 and civil unrest

It is important context that the data presented here were collected at the beginning of 2021. At the time of data collection, several external factors had the potential to impact mental health including the global COVID-19 pandemic, civil unrest, and a divisive political climate. A poll of United States citizens in June of 2020 showed that 4 in 10 adults had symptoms of anxiety or depressive disorder, elevated from 1 in 10 adults in the same period of 2019 (38). Additionally, this survey found that essential workers were at increased risk when compared to the general public for mental health effects of the pandemic, including suicidal ideation (22% vs. 8%) (38). Veterinary professionals in clinically facing roles were deemed essential workers in most jurisdictions of the United States (39).

The aim of the current study was to identify psychological distress, suicidal thoughts, and suicidal behavior in a sample that had the opportunity to disclose diverse demographic identities. Our research questions were:

1. What is the relationship between demographic factors in veterinary professionals, psychological distress, and suicidal experiences?
2. What demographic factors put veterinary professionals most at risk for psychological distress and suicidal experiences?

## Materials and methods

A cross-sectional web-based questionnaire was designed in the Qualtrics survey platform (40) to assess the prevalence of diversity, psychological distress, and suicidality. The survey was distributed across social media channels and emails among various veterinary organizations (Table 1). Data were collected between February 10, 2021, and April 6, 2021, with weekly invitations to participate on social media and listservs (see Table 1). The veterinary organizations that received invitations to participate ranged in size from 491 members to 38,000. To participate in the survey, individuals needed to work or study in the veterinary field in the United States and be 18 or older. This study was acknowledged as exempt by the University of Tennessee Institutional Review Board. As this survey was distributed through social media, the “prevent indexing” Qualtrics feature was enabled to block web engines from listing the survey in search results. Also, any completed response that took less than 10 min was removed from the survey. In addition, respondent data was manually checked for batch entries.

**Table 1:** Organizations that received recruitment invitations.

Organization
American Association of Veterinary Medical Colleges (AAVMC) Association of Asian Veterinary Medical Professionals (AAVMP) Black DVM Network DVM Moms Intersectional discussion Lab Coat/No Closet LGBTQIA Veterinary Professionals Latinx Veterinary Medical Association (LVMA) Moms with a DVM—Life in the Trenches Multicultural Veterinary Medical Association (MCVMA) National Association for Black Veterinarians (NABV) National Association of Veterinary Anesthesia Society National Association of Veterinary Technicians in America (NAVTA) Not One More Vet Public Facing Not One More Vet Student Not One More Vet Support Staff Not One More Vet Veterinarians POC DVM Community Pride Student Veterinary Medical Community VMC Pride Veterinary Medical Community Student American Veterinary Medical Association (SAVMA) Under the Microscope Veterinarian for Veterinarian Veterinarians as One Inclusive Community for Empowerment (VOICE) Veterinary Medical Association Executives (VMAE) Vet to Vet Vets with Disabilities/Chronic Disease Women’s Veterinary Leadership Development Initiative (WVLDI)

### Survey

The demographic portion of the survey questions included race, ethnicity, marital status, social class (poor, working class, middle class, upper middle class, affluent), gender, sexual orientation, disability status, income, and age. Additionally, job-related questions such as role and setting were asked. These demographic questions were self-report questions. Some questions had “yes/no/prefer not to answer” (e.g., Are you currently experiencing a disability, impairment or significant disease?) whereas others listed categories. For example, for annual income, there was a list of income ranges to choose from (e.g., under $25,000, $25,001–50,000, etc.). The survey also included mental health measures of mental well-being, psychological distress, and suicidality.

### Mental health outcomes

#### Psychological distress

The Kessler 6 (K6) (41) measures psychological distress and has been used in multiple studies that assess risk in the veterinary community (12, 18, 23, 24). K6 asks questions based on a Likert scale [e.g., during the past 30 days, about how often did you feel hopeless? All of the time (4), most of the time (3), some of the time (2). A little of the time (2), or none of the time (1)]. The scores are then added together to get a total sum score between 0–24 (41). As the score increases, the level of distress increases. Scores of 13 or higher indicate serious risk of psychological distress. A score between 5–12 indicates risk of moderate psychological distress. A score of 4 or lower indicates low psychological distress. In the current study the reliability of K6 scores as estimated by coefficient alpha was 0.95. In the current study the focus was on scores of 13 or higher, indicative of serious risk of psychological distress. A dichotomous variable was created indicating whether a respondent’s K6 score was equal to or greater than 13 (1 = yes, 0 = no). A dependability index for this classification, equivalent to the Brennan–Kane dependability index, was computed (42). This index for this study was 0.95.

#### Suicidal thinking and behaviors

Suicidal thinking and behaviors were assessed using dichotomous questions that asked about the presence of suicidal thoughts, plans and attempts in the last 12 months (e.g., at any time in the past 12 months, up to and including today, did you seriously think about trying to kill yourself?). Asking about suicidal thinking and behaviors within the “past 12 months” is a common practice in large population-based studies such as the National Survey on Drug Use and Health (NSDUH). The NSDUH has asked these questions since 2015 and have multiple large data sets in which suicide risk has been analyzed (43). For example, a recent study used the 2018 wave to identify suicide risk in sexual minority adults and found an increased risk for suicidal thinking and behaviors compared to heterosexual populations (43).

### Data analysis

Descriptive statistics were used to provide a depiction of the characteristics of the sample in the current study. Two main research questions were investigated in binary logistic regression analyses: (1) what demographic characteristics are associated with severe psychological distress, as indicated by K6 scores of 13 or higher; and (2) what demographic characteristics, over and above severe psychological distress, are associated with suicidal thinking? In these logistic regression analyses, the independent variables were age, gender identification, sexual orientation, marital status, ethnicity, race, practice type, professional role, disability status, social class, number of children in respondent’s family, and total annual income. The dependent variable was respondents answer to the following question, “at any time in the past 12 months, up to and including today, did you seriously think about trying to kill yourself?,” with “yes” coded as 1, and “no” coded as 0.

Given the large number of independent variables, type I error control was important. Thus, tests of the statistical significance of individual independent variables were not conducted unless the overall logistic regression model was statistically significant at the 0.0001 level. Further, specific comparisons of sub-categories of categorical variables, such as comparing sexual orientations of heterosexual with gay/lesbian, were only made if the overall categorical variable was statistically significant. The logistic regression analyses were conducted using Mplus version 7.4, and missing data were managed using full information maximum likelihood (FIML). Robust maximum likelihood estimation was used.

Some subcategories of categorical variables had small numbers of cases, which is often handled by combining categories because of methodological issues associated with small sample sizes and sparse data (44). In the current study this was not done given the important objective of inclusion of all forms of diversity in the data analyses. To help control for problems incurred by small sample sizes in subcategories, 99% confidence intervals were used to help facilitate interpretations of results involving subcategories with small sample sizes.

## Results

### Participants

Of 2,482 responses, 2,208 had usable data and were included in this analysis. Demographic data are reported in Tables 2–4. Eleven percent of respondents identified as African/Black, Indigenous, Asian, Pacific Islander, Middle Eastern, or two or more races (*N* = 235). Two percent (*N* = 43) indicated their race was not listed, and 1% (*N* = 22) chose not to answer. Regarding gender, 11% (*N* = 247) of our sample identified as transgender, gender non-conforming, gender fluid, agender, questioning, or other, and 3% (*N* = 69) chose not to answer. With sexual orientation, 22% (*N* = 448) identified as gay/lesbian, bi+/non-monosexual, fluid, queer, questioning, asexual, or other, and 1% (*N* = 18) chose not to answer. Regarding relationship status, 39% (*N* = 865) indicated they are separated, divorced, widowed, or never married. 67% (*N* = 1,474) do not have any children. Ten percent of the sample is between the ages of 18–24 (*N* = 227), and 7% are above the age of 55 (*N* = 154). When asked about the presence of a current disability, disease, or significant illness, 20% (*N* = 443) indicated yes. Two percent preferred not to answer (*N* = 51). Three percent of the participants identified as poor (*N* = 59), and 30% identified as working class (*N* = 645). Regarding annual income, 9% of the sample indicated their household income is less than $25,000 a year (*N* = 187), and 20% reported between $25,000–$50,000 (*N* = 433). Finally, 63% of the respondents were employed as veterinary professionals (customer service, unlicensed veterinary assistant, credentialed technician, management, or other), and 37% were veterinarians.

**Table 2:** Participant characteristics—gender, age, race, ethnicity, sexual orientation and disability status.

Variable	No. (%) of respondents	K6 (serious)	Past 12 consider	Plan	Attempt
Overall sample	**2,208**	888 (40.2%)	381 (17.3%)	88 (4.0%)	16 (0.7%)
*Gender*					
Cisgender female	1,760 (79.7%)	702 (39.9%)	288 (16.4%)	72 (4.1%)	15 (0.9%)
Cisgender male	109 (4.9%)	36 (33.0%)	13 (11.9%)	5 (4.6%)	1 (0.9%)
Transgender female	73 (3.3%)	34 (46.6%)	16 (21.9%)	1 (1.4%)	0 (0.0%)
Transgender male	11 (0.5%)	4 (36.4%)	4 (36.4%)	0 (0.0%)	0 (0.0%)
Gender non-conforming	33 (1.5%)	22 (66.7%)	11 (33.3%)	2 (6.1%)	0 (0.0%)
Gender fluid	17 (0.8%)	12 (70.6%)	9 (52.9%)	1 (5.9%)	0 (0.0%)
Agender	7 (0.3%)	1 (14.3%)	3 (42.9%)	1 (14.3%)	0 (0.0%)
Questioning	12 (0.5%)	6 (50.0%)	4 (33.3%)	1 (8.3%)	0 (0.0%)
Other	94 (4.3%)	31 (33.0%)	18 (19.1%)	4 (4.3%)	0 (0.0%)
Prefer not to answer	69 (3.1%)	36 (52.2%)	15 (21.7%)	1 (1.4%)	0 (0.0%)
Missing	23 (1.0%)	0 (0.0%)	0 (0.0%)	0 (0.0%)	0 (0.0%)
*Age*					
18–24	227 (10.3%)	141 (62.1%)	81 (35.7%)	12 (5.3%)	2 (0.9%)
25–34	881 (39.9%)	400 (45.4%)	190 (21.6%)	46 (5.2%)	13 (1.5%)
35–44	626 (28.6%)	233 (37.2%)	84 (13.4%)	23 (3.7%)	1 (0.2%)
45–54	299 (13.5%)	76 (25.4%)	15 (5.0%)	3 (1.0%)	0 (0.0%)
55–64	125 (5.7%)	32 (25.6%)	6 (4.8%)	2 (1.6%)	0 (0.0%)
65–74	25 (1.1%)	4 (16.0%)	3 (12.0%)	2 (8.0%)	0 (0.0%)
75+	4 (0.2%)	2 (50.0%)	1 (25.0%)	0 (0.0%)	0 (0.0%)
Missing	21 (1%)	0 (0.0%)	1 (4.8%)	0 (0.0%)	0 (0.0%)
*Race*					
White	1,881 (85.2%)	756 (40.2%)	321 (17.1%)	74 (3.9%)	13 (0.7%)
African Diaspora	21 (1%)	11 (52.4%)	1 (4.8%)	0 (0.0%)	0 (0.0%)
Indigenous	23 (1.1%)	10 (43.5%)	7 (30.4%)	0 (0.0%)	0 (0.0%)
Asian	86 (3.9%)	32 (37.2%)	11 (12.8%)	2 (2.3%)	0 (0.0%)
Pacific Islanders	2 (0.1%)	0 (0.0%)	0 (0.0%)	0 (0.0%)	0 (0.0%)
Middle Eastern	7 (0.3%)	5 (71.4%)	1 (14.3%)	0 (0.0%)	0 (0.0%)
Two or more races	96 (4.3%)	41 (42.7%)	21 (21.9%)	5 (5.2%)	2 (2.1%)
Prefer not to answer	22 (1.0%)	7 (31.8%)	3 (13.6%)	1 (4.5%)	0 (0.0%)
Not listed	43 (1.9%)	24 (55.8%)	15 (34.9%)	6 (14.0%)	1 (2.3%)
Missing	4 (0.2%)	0 (0.0%)	0 (0.0%)	0 (0.0%)	0 (0.0%)
Ethnicity*			(0.0%)	(0.0%)	(0.0%)
Hispanic	133 (6%)	62 (46.6%)	34 (25.6%)	8 (6.0%)	1 (0.8%)
Other Hispanic	22 (1.0%)	7 (31.8%)	2 (9.1%)	2 (9.1%)	0 (0.0%)
Non-Hispanic	1,942 (88.0%)	789 (40.6%)	332 (17.1%)	74 (3.8%)	15 (0.8%)
Prefer not to answer	48 (2.2%)	18 (37.5%)	9 (18.8%)	3 (6.3%)	0 (0.0%)
Missing	63 (2.9%)	12 (19.0%)	4 (6.3%)	1 (1.6%)	0 (0.0%)
*Sexual orientation*					
Heterosexual	1,692 (76.6%)	630 (37.2%)	234 (13.8%)	56 (3.3%)	11 (0.7%)
Homosexual	86 (3.9%)	35 (40.7%)	14 (16.3%)	2 (2.3%)	0 (0.0%)
Bi/Non-monosexual	275 (12.5%)	157 (57.1%)	100 (36.4%)	26 (9.5%)	4 (1.5%)
Fluid	13 (0.6%)	6 (46.2%)	5 (38.5%)	0 (0.0%)	0 (0.0%)
Queer	32 (1.4%)	13 (40.6%)	7 (21.9%)	1 (3.1%)	0 (0.0%)
Questioning	27 (1.2%)	18 (66.7%)	8 (29.6%)	0 (0.0%)	0 (0.0%)
Other	11 (0.5%)	4 (36.4%)	4 (36.4%)	1 (9.1%)	0 (0.0%)
Prefer not to answer	18 (0.8%)	7 (38.9%)	1 (5.6%)	0 (0.0%)	0 (0.0%)
Asexual	34 (1.5%)	16 (47.1%)	8 (23.5%)	2 (5.9%)	1 (2.9%)
Missing	20 (0.9%)	2 (10.0%)	0 (0.0%)	0 (0.0%)	0 (0.0%)
*Disability status*					
Yes	443 (20.1%)	217 (49.0%)	99 (22.3%)	26 (5.9%)	5 (1.1%)
No	1,671 (75.7%)	637 (38.1%)	266 (15.9%)	57 (3.4%)	8 (0.5%)
Prefer not to answer	51 (2.3%)	28 (54.9%)	14 (27.5%)	4 (7.8%)	2 (3.9%)
Missing	43 (1.9%)	6 (14.0%)	2 (4.7%)	1 (2.3%)	1 (2.3%)

^*^Ethnicity was categorized in the survey as follows: (1) Hispanic (Argentinian, Bolivian, Mexican, Spanish, Columbian, Cuban, Dominican, Ecuadorian, Puerto Rican, etc.) (2) Other Hispanic, Latinx, or Spanish Origin (3) Non-Hispanic (4) Prefer not to answer. Overall sample size is bolded.

**Table 3:** Participant characteristics–marital status, children, income and social class.

Variable	No. (%) of respondents	K6 (serious)	Past 12 consider	Plan	Attempt
Overall sample	**2,208**	**888 (40.2%)**	**381 (17.3%)**	**88 (4.0%)**	**16 (0.7%)**
*Marital status*					
Married/committed	1,327 (60.1%)	480 (36.2%)	208 (15.7%)	53 (4.0%)	9 (0.7%)
Separated	32 (1.4%)	17 (53.1%)	11 (34.4%)	1 (3.1%)	1 (3.1%)
Divorced	144 (6.5%)	57 (39.6%)	15 (10.4%)	4 (2.8%)	0 (0.0%)
Widowed	14 (0.6%)	3 (21.4%)	0 (0.0%)	0 (0.0%)	0 (0.0%)
Never Married	675 (30.6%)	331 (49.0%)	147 (21.8%)	30 (4.4%)	6 (0.9%)
Missing	16 (0.8%)	(0.0%)	0 (0.0%)	0 (0.0%)	0 (0.0%)
*Number of children*					
0	1,474 (66.8%)	665 (45.1%)	297 (20.1%)	61 (4.1%)	11 (0.7%)
1	255 (11.5%)	92 (36.1%)	42 (16.5%)	14 (5.5%)	2 (0.8%)
2	301 (13.6%)	87 (28.9%)	25 (8.3%)	7 (2.3%)	0 (0.0%)
3	109 (4.9%)	28 (25.7%)	12 (11.0%)	4 (3.7%)	2 (1.8%)
4 or more	49 (2.2%)	15 (30.6%)	5 (10.2%)	2 (4.1%)	1 (2.0%)
Missing	20 (0.9%)	1 (5.0%)	0 (0.0%)	0 (0.0%)	0 (0.0%)
*Income*					
25 k	187 (8.5%)	(0.0%)	54 (28.9%)	13 (7.0%)	1 (0.5%)
25–50	433 (19.6%)	244 (56.4%)	141 (32.6%)	34 (7.9%)	7 (1.6%)
50–75	289 (13.1%)	141 (48.8%)	70 (24.2%)	14 (4.8%)	3 (1.0%)
75–100	321 (14.5%)	130 (40.5%)	44 (13.7%)	9 (2.8%)	2 (0.6%)
100–150	392 (17.8%)	(0.0%)	22 (5.6%)	5 (1.3%)	0 (0.0%)
150–200	207 (9.4%)	37 (17.9%)	10 (4.8%)	1 (0.5%)	0 (0.0%)
200 or more	212 (9.6%)	42 (19.8%)	10 (4.7%)	4 (1.9%)	0 (0.0%)
Prefer not to answer	121 (5.5%)	57 (47.1%)	28 (23.1%)	7 (5.8%)	2 (1.7%)
Missing	46 (2.1%)	6 (13.0%)	2 (4.3%)	1 (2.2%)	1 (2.2%)
*Social class*					
Poor	59 (2.7%)	41 (69.5%)	23 (39.0%)	5 (8.5%)	0 (0.0%)
Working class	645 (29.2%)	374 (58.0%)	195 (30.2%)	44 (6.8%)	10 (1.6%)
Middle class	844 (38.2%)	321 (38.0%)	123 (14.6%)	27 (3.2%)	3 (0.4%)
Upper middle class	536 (24.3)	122 (22.8%)	28 (5.2%)	7 (1.3%)	0 (0.0%)
Affluent	57 (2.6%)	11 (19.3%)	4 (7.0%)	2 (3.5%)	0 (0.0%)
Prefer not to answer	23 (1.0%)	13 (56.5%)	6 (26.1%)	2 (8.7%)	2 (8.7%)
Missing	44 (2%)	6 (13.6%)	2 (4.5%)	1 (2.3%)	1 (2.3%)

Overall sample size is bolded.

**Table 4:** Participant characteristics—professional role.

Variable	No. (%) of respondents	K6 (serious)	Past 12 consider	Plan	Attempt
*Overall sample*	**2,208**	**888 (41%)**	**381 (17.3%)**	**88 (4.0%)**	**16 (0.7%)**
*Professional role*					
Customer service	74 (3.4%)	40 (54.1%)	26 (35.1%)	5 (6.8%)	4 (5.4%)
Unlicensed vet assist	237 (10.7%)	137 (57.8%)	79 (33.3%)	20 (8.4%)	6 (2.5%)
Credentialed tech	554 (25.1%)	266 (48.0%)	169 (30.5%)	36 (6.5%)	4 (0.7%)
Management associate	100 (4.5%)	41 (41.0%)	23 (23.0%)	6 (6.0%)	1 (1.0%)
Associate veterinarian	562 (25.5%)	161 (28.6%)	0 (0.0%)	0 (0.0%)	0 (0.0%)
Relief vet	78 (3.5%)	19 (24.4%)	0 (0.0%)	0 (0.0%)	0 (0.0%)
Practice owner	183 (8.3%)	51 (27.9%)	0 (0.0%)	0 (0.0%)	0 (0.0%)
Intern/resident	54 (2.4%)	18 (33.3%)	12 (22.2%)	1 (1.9%)	0 (0.0%)
Student	198 (9.0%)	98 (49.5%)	51 (25.8%)	12 (6.1%)	1 (0.5%)
Other	153 (6.9%)	57 (37.3%)	30 (19.6%)	8 (5.2%)	1 (0.7%)
Missing	15 (0.7%)	0 (0.0%)	0 (0.0%)	0 (0.0%)	0 (0.0%)

Overall sample size is bolded.

Tables 2–4 also indicates the overall at-risk results of the K6, and suicidal experiences. 41% (*N* = 888) of the sample met the criteria for serious psychological distress. For suicide experiences, 17.3% (*N* = 381) of the sample considered suicide, 4% (*N* = 88) planned suicide, 0.7% (*N* = 16) attempted suicide, 0.4% (*N* = 9) received medical attention from an attempt, and 0.3% (*N* = 7) were hospitalized in the past 12 months due to injury from suicide attempt. Tables 2–4 report each demographic category and the percent within each category that had high K6 scores or answered yes on the suicide thinking/behavior questions. For example, Table 2 reports that 16.4% (*N* = 288) of all cisgendered female survey respondents (*N* = 1,760) selected “yes” when asked if they had considered suicide within the past 12 months.

### Results of descriptive statistics

Of the overall sample, 888 respondents (40.2%) were experiencing serious psychological distress. In the gender category, cisgender males had the lowest percentage of respondents experiencing psychological distress (*N* = 36, 33.0%). The highest were those who identified as gender fluid (*N* = 12, 70.6%) and gender non-conforming (*N* = 26, 66.7%). However, all of these sample sizes were small. The categories with the highest percentage of those that considered suicide in the past 12 months were agender (*N* = 3, 42.9%), transgender male (*N* = 4, 36.4%), gender non-conforming (*N* = 11, 33.3%) and questioning (*N* = 4, 33.3%). In the largest gender category, cisgender female, the percentage of serious psychological distress was 39.9% (*N* = 702). For the age category, the younger the participant, the more likely they were to have serious psychological distress. For example, in the age range of 45–54 (*N* = 76), 25.4% had high K6 scores whereas in the 18–24 years category, 62.1% did (*N* = 141). Those in the youngest age bracket also had the highest percentage of respondents who had considered suicide in the past year (*N* = 81, 35.7%).

For race, the categories that experienced the highest rates of psychological distress was Middle Eastern (*N* = 5, 71.4%), African Diaspora (*N* = 11, 52.4%), Indigenous (*N* = 10, 43.5%), and two or more races (*N* = 41, 42.7%). Each of these categories had small sample sizes however, requiring caution in interpreting. For those who identified with the largest race category, White, the percentage experiencing serious K6 scores was 40.2%. For ethnicity, the category with the largest percentage experiencing serious psychological distress was Hispanic (*N* = 62; 46.6%). The categories that had the highest percentage of those that considered suicide in the past year were Indigenous (*N* = 7, 30.4%) and two or more races (*N* = 21, 21.9%).

In the sexual orientation category, those who identified as questioning had the highest rate of serious distress (*N* = 18, 66.7%). They were followed by bisexual (*N* = 157, 40.7%), asexual (*N* = 16, 47.1%), and fluid (*N* = 6, 46.2%). With the exception of the bisexual category, all of the other above categories had a small sample size. Those who identified as heterosexual had the lowest rate of psychological distress (*N* = 630, 37.2%). Five respondents who identified as fluid (38.5%) and one hundred respondents who identified as bisexual (36.4%) indicated they considered suicide in the past year.

For disability status, those who indicated they were currently experiencing a disability, had a higher percentage experiencing serious psychological distress (*N* = 217, 49.0%) over those who were not experiencing a disability (*N* = 637, 38.1%). The percentage of those with a current disability that considered suicide in the past 12 months was 22.3% (*N* = 99). In the marital status category, those who selected the response of separated had the highest K6 scores (*N* = 17, 53.1%) followed by never married (*N* = 331, 49.0%). The lowest rate of K6 scores for marital status was the widowed (*N* = 3, 21.4%) and in a married or committed relationship (*N* = 480, 36.2%). For the number of children, those who selected “0” children had the highest K6 scores (*N* = 665, 45.1%). The lowest rate of serious psychological distress in the children category was for those who have three children (*N* = 28, 25.7%). Again, small sample sizes (e.g., having 3 children) needs to be interpreted with caution. The categories that had the highest percentage of those who considered suicide in the last 12 months were also those who were separated (*N* = 11, 34.4%), never married (*N* = 147, 21.8%), did not have any children (N = 297, 20.1%) or had one child (*N* = 42, 16.5%).

In the demographic category of annual income, those who selected $25,000–50,000 had the highest K6 scores (*N* = 244, 56.4%). In general, as the income bracket increased, the rates of serious psychological distress decreased. This same trend was true for social class. For those who selected “poor” social class, 69.5% had high K6 scores (*N* = 41). For those who selected an “affluent” social class, 19.3% had high K6 scores (*N* = 11). For those that considered suicide in the past 12 months, the highest percentages were in the income bracket of under $25,000 (*N* = 54. 28.9%), $25,000–500,000 (*N* = 141, 32.6%), and $50,000–75,000 (*N* = 70, 24.2%), poor social class (*N* = 23, 39%), and working class (*N* = 195, 30.2%).

With professional roles, those who selected unlicensed veterinarian assistants had the highest rates of serious K6 scores (*N* = 137, 57.8%). This was followed by customer service (*N* = 40, 54.1%) and student (*N* = 98, 49.5%). Practice owners had the lowest percentage of serious psychological distress (*N* = 51, 27.9%) followed by associate veterinarians (*N* = 161, 28.6%). This trend is similar for those that considered suicide in the past 12 months. Those who identified as having a customer service or unlicensed veterinarian assistant role had the highest percentages of considering suicide (*N* = 26, 35.1% and *N* = 79, 33.3% respectively).

### Results of logistic regression analyses

#### Results for K6 level

The overall chi-square for this model was, *χ*^2^(61) = 285.79, *p* < 0.0000001. Statistically significant chi-square values for specific variables were for gender, *χ*^2^(9) = 17.74, *p* = 0.038; social class, *χ*^2^(5) = 52.59, *p* < 0.0001; age, *χ*^2^(1) = 10.47, *p* = 0.0012; number of children, *χ*^2^(1) = 4.82, *p* = 0.028; and disability, *χ*^2^(1) = 11.04, *p* = 0.0009. These were statistically significant predictors of significant levels of K6. Professional role, *χ*^2^(8) = 9.16, *p* = 0.33; sexual orientation, *χ*^2^(8) = 13.33 *p* = 0.10; professional practice, *χ*^2^(14) = 16.5, *p* = 0.28; marital status, *χ*^2^(4) = 4.58, *p* = 0.33; annual income, *χ*^2^(1) = 0.83, *p* = 0.36; race, *χ*^2^(6) = 3.17, *p* = 0.79; and ethnicity, *χ*^2^(3) = 4.53, *p* = 0.21, were statistically nonsignificant predictors of K6 level. The R^2^ for this model was 0.19, *z* = 9.82, *p* < 0.001; about 19% of the total variation in K6 scores was explained by the independent variables. In terms of area under the curve (AUC), this effect size is 0.75.

The specific results for the statistically significant predictors are shown in Table 5. Statistically nonsignificant results are not included in this table in the interest of space and brevity.

**Table 5:** Results for predictors of K6 level.

Variable	*B*	*z*	*p*	Odds ratio	99% CI odds ratio
Number of children	−0.12	−2.20	0.028	0.89	0.77, 1.02
*Social class*					
Poor (*n* = 59) compared with middle class (*n* = 844)	1.12	3.36	0.001	3.06	1.30, 7.24
Working class (*n* = 645) compared with middle class	0.588	4.75	<0.001	1.80	1.31, 2.48
Upper middle class (*n* = 563) compared with middle class	−0.53	−3.80	<0.001	0.59	0.41, 0.84
*Gender*					
Gender nonconforming (*n* = 33) compared with cisgender female (*n* = 1,760)	1.003	2.44	0.015	2.80	0.95, 7.85
Disability (*n* = 443) compared with no disability (*n* = 1,721)	0.40	3.32	0.001	1.49	1.09, 2.03
Age	−0.02	−3.23	0.001	0.96	0.966, 0.996

These results strongly suggested that social class (0.0001 level of statistical significance), age (0.001 level of statistical significance) and disability (0.001 level of statistical significance) were associated with significant K6 scores. Specifically, the odds of those identifying as being poor were about three times greater than those identifying as in the middle class to have significant K6 scores. Those identifying as working class were about 1.8 times more likely than those identifying as middle class to have significant K6 scores. Those identifying as upper middle class were slightly more than half as likely to have significant K6 scores than those identifying as middle class. Those reporting some form of disability were nearly one-and-a-half time more likely to have significant K6 scores as those not reporting any disability. As the age of respondents increased, the odds they had significant K6 scores decreased (odds ratio = 0.96 for each year age increased).

Results also suggested, at the 0.05 level of statistical significance, that as the numbers of children a respondent had increased, the less likely they were to have significant K6 scores (odds ratio = 0.89 per each child). Those self-identifying as gender nonconforming were more likely than those identifying as cis-gender female to have significant K6 scores (odds ratio = 2.80), though these specific results were based on a very small sample of respondents identifying as gender nonconforming (*n* = 33), so these results should be interpreted cautiously and as tentative.

The categorical variable “sexual orientation” as a whole was statistically non-significant, *χ*^2^(8) = 13.3, *p* = 10. But the Bi+ category regression coefficient was statistically significant, *b* = 0.44, *z* = 2.92, *p* = 0.003.

### Results for suicidal thinking with K6 level as a predictor

The overall chi-square for this model was, *χ*^2^(62) = 334.42, *p* < 0.0000001. Sexual orientation, *χ*^2^(8) = 29.86, *p* = 0.0002; marital status, *χ*^2^(4) = 10.82, *p* = 0.029; professional role, *χ*^2^(8) = 17.94, *p* = 0.022; and K6, *χ*^2^(1) = 185.3, *p* < 0.0001 were statistically significant predictors of suicidal thinking. Race, *χ*^2^(6) = 9.74, *p* = 0.14; gender, *χ*^2^(9) = 2.33, *p* = 0.98; professional practice role, *χ*^2^(14) = 16.42, *p* = 0.29; ethnicity, *χ*^2^(3) = 0.58, *p* = 0.90; annual income, *χ*^2^(1) = 0.82, *p* = 0.36; age, *χ*^2^(1) = 3.35, *p* = 0.07; disability, *χ*^2^(1) = 2.13, *p* = 0.14; social class, *χ*^2^(5) = 2.25, *p* = 0.81; and number of children, *χ*^2^(6) = 0.50, *p* = 0.48 were statistically nonsignificant. The *R*^2^ for this analysis was 0.27, *z* = 11.73, *p* < 0.001; this model accounted for 27% of the variation in suicidal thinking scores. This effect size is 0.80 in terms of AUC.

The specific results for statistically significant predictors are shown in Table 6. Statistically nonsignificant results are not included in this table in the interest of space and brevity.

**Table 6:** Results for predictors of suicidal thinking, with K6 level as a predictor.

Variable	*B*	*z*	*p*	Odds ratio	99% CI odds ratio
*Sexual orientation*					
Bi+ (monosexual, bisexual, pansexual, omnisexual; *n* = 275) compared to heterosexual (*n* = 1,692)	0.76	4.60	<0.001	2.14	1.39, 3.29
Fluid (*n* = 13) compared to heterosexual	1.10	2.37	0.014	3.00	0.90, 10.00
*Marital status*					
Separated (*n* = 32) compared with married/in a committed relationship (*n* = 1,327)	1.04	2.42	0.015	2.83	0.94, 8.58
K6	1.68	13.61	<0.0001	5.37	3.92, 7.39
*Professional role*					
Relief vet (*n* = 78) compared with associate vet (*n* = 562)	0.80	2.58	0.01	2.22	1.001, 4.90
“Other role” (*n* = 153) compared with associate vet	−0.69	−2.40	0.016	0.50	−0.24, 1.05

These results suggested at the 0.001 level of statistical significance that those self-identifying as monosexual, bisexual, pansexual, or omnisexual were more than twice as likely to report suicidal thinking, holding K6 level constant, than those self-identifying as heterosexual. If this sexual orientation is compared with all others, except gender fluid (see following), the odds a respondent identifying as monosexual, bisexual, pansexual, or omnisexual remain twice as likely as any of the other sexual orientations to report suicidal thinking (odds ratio = 2.02, *p* < 0.001).

A weaker finding, based on only 13 respondents, hinted that those identifying as gender fluid may be as much as 3 times more likely than those identifying as heterosexual to report suicidal thinking. If gender fluid is compared with all other sexual orientations (except Bi+), the odds drop to slightly more than 2 times that those identifying as gender fluid will report suicidal thinking as will those identifying as any of the other sexual orientations (except for Bi+). This finding, given the very small sample size involved, should be taken as suggestive and interpreted within the context of the very small sample size of *n* = 13.

Results also suggested, at the 0.05 level of statistical significance, that persons separated from a spouse or partner may be nearly 3 times more likely to report suicidal thinking than those married or in a committed relationship. This finding is based on a small sample of persons separated (n = 32), so these findings should be taken as suggestive and tentative.

Results also suggested, at the 0.01 level of statistical significance, that those in the role of a relief vet were about 2.2 times more likely to report suicidal thinking than those in the role of an associate vet. This result should be interpreted cautiously as the sample of respondents identifying as relief vets was only 78. Results further suggested, at the 0.02 level of statistical significance, that those in “other” professional roles were only about half as likely to report suicidal thinking as those in the role of associate vet. If these professional roles are compared with all other professional roles, those in the role of relief vet were about 2 times more likely (*p* = 0.017), and those in “other” professional roles less than half as likely (odds ratio = 0.44, *p* = 0.003), as those in any other professional roles to report suicidal thinking, holding K6 level constant.

Finally, results strongly suggested, at the 0.0001 level of statistical significance, that respondents with significant K6 scores were more than 5 times more likely to report suicidal thinking than those with nonsignificant K6 scores.

### Kessler 6 and suicidal thoughts

The younger the participant [*X*^2^(6, *N* = 2,158) = 99.579, *p* < 0.001] and the lower social class [*X*^2^(5, *N* = 2,142) = 185.903, *p* < 0.001] they selected, the more likely they were to experience psychological distress. Compared to married participants, those who were separated were more likely to experience serious distress [X^2^(4, *N* = 2,162) = 36.654, *p* < 0.001]. Compared to those who did not identify as disabled, those with disabilities were more likely to have a higher K6 score [*X*^2^(2, *N* = 2,142) = 20.594, *p* < 0.001]. In the gender category, those who identified as questioning were less likely to meet threshold K6 levels than cisgender women [X^2^(9, *N* = 2,155) = 28.730, *p* < 0.001]. Bisexual+/non-monosexual individuals were also more likely to have serious psychological distress when compared to heterosexual individuals [*X*^2^(16, *N* = 2,157) = 62.910, *p* < 0.001]. For those more likely to experience a serious psychological distress score, they were also more likely to have lower mental well-being [*X*^2^(1, *N* = 2,053) = 302.940, *p* < 0.001] and more likely to have suicidal thoughts [*X*^2^(1, *N* = 1,343) = 175.743, *p* < 0.001].

### Sexual orientation

A *post hoc* analysis of sexual orientation was performed, merging results into three categories: heterosexual, gay/lesbian, and bisexual/other (fluid, queer, questioning, asexual). Participants who identified as bisexual, fluid, queer, questioning, asexual, or other were more likely to have serious psychological distress and considered suicide in the past 12 months (*p* < 0.001). They also were more likely to make a suicide plan in the past 12 months (*p* < 0.05). This *post hoc* analysis used pairwise comparisons using the *z*-test of two proportions with a Bonferroni correction (Table 7).

**Table 7:** Sexual orientation chi-square results.

Variable	Hetero (*N* = 1,692)	Gay/lesbian (*N* = 86)	BiSex/Other (*N* = 392)	*X*^2^ (*df*), *p*
K6-serious	630 (37.7%)_a_	35 (40.7%)_a_	214 (55.2%)_b_	39.99 (2), *p* < 0.001
Past 12 consider	234 (23.5%)_a_	14 (29.8%)_a,b_	1,324 (2.7%)_b_	43.06 (2), *p* < 0.001
Past 12 plan	56 (4.1%)_a_	2 (0.1%)_a,b_	30 (9.7%)_b_	6.83 (2), *p* = 0.033
Past 12 attempt	11 (12.4%)_a_	0 (0.0%)_a_	5 (16.7%)_a_	0.51 (2), *p* = 0.763

^a,b^Differing subscript values indicate which groups of the independent variable have different statistically significant proportions (*p* < 0.05), based on chi-square with Bonferroni correction.

## Discussion

There has been growing attention to mental health and suicidal thinking and behaviors in veterinary medicine for the past several years (23, 45). The findings of this research continue to shed light on this topic and affirm that those in the field of veterinary medicine experience both psychological distress as well as suicidal thinking and behaviors. Within context, the 2021 results of the National Survey on Drug Use and Health (NSDUH, 2021), indicate that 22.8% of adults experienced mental disorders and 4.8% experienced suicidal ideation. A meta-analysis of 54 studies exploring suicidal behaviors during the pandemic found 10.8% of respondents experienced suicidal thinking (46). However, estimates for health care workers were higher. For instance, a cross sectional study exploring mental health impacts of COVID among health care workers found 57% reported acute stress, 48% symptoms of depression, and 33% symptoms of anxiety. In another study, 26% of essential workers, which members of the veterinary industry were, reported suicidal thinking (47). Thus our rates of psychological distress and suicidal thinking were also found in other similar populations during the pandemic. Additionally, several demographics were associated with increased risk of psychological distress and suicidal thinking in our findings.

### Demographic identity

#### Gender identity

Cisgender women were more likely than cisgender men to experience psychological distress. Male veterinarians have been shown to have higher well-being scores than their female counterparts in several studies (12, 23). While those analyses did not account for gender identity within their samples, there is evidence in both the veterinary and general literature that cisgender identification is protective for wellbeing (24, 48–50). Those who identified as transgender females, transgender males, gender non-conforming, or gender fluid were more likely to experience serious psychological distress than cisgender male and cisgender females. Of all the gender categories, the only one that showed statistical significance in the logistic regression was gender non-conforming. However, the sample size was very small (*n* = 33) as compared with cisgender female (*n* = 1,760). Though many of the other categories did not reach statistical significance, the above trends as indicated by percentages are in line with much of the literature published on gender identity and psychological distress, including studies within the veterinary community, wherein individuals with minoritized gender identities score lower in various wellbeing measurements and higher in suicidality risk (24, 48, 49). A larger sample size might bring these trends into significance. Within the literature on gender identity and psychological well being, factors such as outness, a sense of belonging and exposure to violence, have been shown to have impact on wellbeing and suicidality in minoritized gender identity groups (51–53). As these variables were not quantified in this dataset and the sample size of non-binary categories, definitive conclusions are difficult to draw.

#### Sexual identity

Heterosexuality was associated with a decreased K6 score and thoughts of suicide in comparison to other groups. The protective relationship of heterosexuality to psychological distress has been well shown in the literature (54–56). Non-heterosexual identities may face a number of challenges to their wellbeing including threats of violence, discrimination, and harassment (24, 51, 55). Previous large studies of veterinary wellbeing and suicidality did not directly compare heterosexuals to other sexual identities (12, 18, 45). However, in a recent analysis, non-heterosexual identities were found to have higher levels of suicidal ideation and attempts, and non-heterosexual women were found to have higher K6 scores than veterinary populations as a whole (24). In the current study, many of the sexual orientation categories had small sample sizes thus have limitations. However, the category of bi-sexual had a sample size of 275 and demonstrated a clear statistical significantly increased risk of serious psychological distress. In addition, identifying as bi-sexual was also significantly associated with a higher risk of suicidal thinking.

Identifying as gay or lesbian, in our study, had a slightly higher percentage of respondents with serious K6 scores. However, this category did not demonstrate statistical significance. This could be because of the small sample size of the gay/lesbian category (*n* = 86). However, in a recent representative study that took place during COVID-19, 65% of those who identified as bi-sexual experienced depression or anxiety, whereas 50% of those who identified as gay or lesbian experienced depression or anxiety. The same trend was true for suicidal ideation. Those who identified as bi-sexual were much more likely (39%) to experience suicidal ideation than those who identified as gay or lesbian (23%) (47).

In a good deal of the literature, Bi/non-monosexual, queer, questioning, and asexual identities are often combined or not represented at all. Even within an umbrella of bisexuality there is a relative lack of inclusion of the bisexual community in psychological literature. In one large scale review of LGBTQ depression and anxiety, 52% of the articles were rejected as they did not report separate data for bisexual people (57). In our *post hoc* analysis, non-gay/lesbian/heterosexual orientations were more likely to have serious psychological distress. They were also more likely to have considered and made a suicide plan in the past 12 months. This is in line with the available data for these communities and mental health (55, 57–62). Poor mental health outcomes within these communities have been largely attributed to erasure/invisibility, a lack of affirming support, as well as double discrimination from both the hetero and LGBTQ+ communities (57, 59–61). These factors contribute to a lack of a supportive community and sense of belonging, which has been shown to negatively impact mental health outcomes in sexual minorities (24, 59, 60).

#### Social class

The results of this research indicate social class was associated with psychological distress. The lower the social class, the higher the percentage of respondents who met criteria for serious psychological distress. Those who identified in the poor, working class, or upper middle class (compared with middle class) were statistically more likely to experience serious psychological distress. Financial problems have a high association with psychological distress and mental well-being (63, 64). Because role is often associated with social class, individuals who seem to carry more professional roles and have higher socio-economic status have better mental health than those who are in roles of direct labor (65, 66). Even so, health care workers with higher levels of socio-economic status experience more serious psychological distress than the general public (67).

#### Marital status

For relationship status, those who indicated they were separated had statistically increased K6 scores and were more likely to have suicidal thoughts in the last 12 months than those who were married or for those that were single. Some literature indicates that marriage is a protective factor (68) for psychological distress whereas some literature indicates that marriage is a risk factor (37). Considering the context of the COVID-19 pandemic during this study, it is worth noting that divorce rates appeared to be increasing early in the pandemic, but analyses from 2020 and 2021 indicate that overall divorce rates slowed in the United States (69). Relationship scientists indicate that couples may be greatly affected by (1) the uncertainties and stressors of the pandemic and (2) the underlying pre-existing vulnerabilities in their relationship pre-pandemic (70).

#### Disability

Currently experiencing a disability/limitation statistically increased K6 and whether or not an individual considered suicide. The relationship between disability status and psychological distress is well represented in the literature. Those who have a disability may experience serious psychological distress at a rate of seven times more than those who do not have a disability (71). Historically, individuals with disabilities were not welcome in veterinary medicine as they were seen as unable to comply with the high demands of the field (72). Odunayo and Zenithson argue that when those with disabilities are provided with appropriate accommodations, they enrich the field of veterinary medicine (73). Veterinary scholars suggest increasing diversity, equity, and inclusion training in clinics and professional organizations to include disability topics (73, 74).

#### Age

Age is consistently found as protective for mental health concerns in veterinary populations (45, 75–77). This is in line with findings in the general population that mental health improves with age. During 2021 NSDUH findings indicate that 33.7% of 18–25 years-olds had psychological distress, 21.8% of those 26–49, and 15% of those older than 50 reported psychological distress (NSDUH, 2021). These findings are likely related to improved emotional regulation, communication and coping skills developed over time (78). In our findings age also significantly predicted serious psychological distress-specifically as age went up distress went down.

#### Race

Surprisingly, in this study, race was not a statistical predictor of psychological distress, suicidal thinking, or suicidal behaviors. However, when looking at the descriptive statistics, there are a higher percentage of respondents who have high K6 scores if they selected their race as African American/Black, Indigenous, Middle Eastern, or two or more races than for those who selected their race as white. Although the relationship between experiences with race and psychological distress is documented in the medical profession (79, 80), there is a lack of studies on the connection between race/ethnicity and psychological distress, suicidal thoughts or suicidal behaviors in the veterinary profession. For our study, likely due to large differences in sample size between diverse race and ethnic diversity and white participants, the analysis was not able to make any statistically meaningful inferences. Further studies need to be conducted to identify a clearer picture of the relationship between race/ethnicity and psychological distress in the veterinary profession.

#### COVID-19 influence

Data collection for this analysis occurred during the COVID-19 pandemic. Negative mental health and wellbeing impacts of the pandemic have been noted on a global scale (48, 81). Specific challenges in the veterinary community have included increased exposure to ethically challenging situations, increased workload, and decreased staffing (12, 82). Impacts on wellbeing and mental health are mixed in the literature. An analysis of US veterinarians showed no change in veterinary well-being, nor in the prevalence of suicidal ideation or attempts, compared to their 2019 analysis (45). However, this same analysis did note a significant increase in serious psychological distress (45) and declines in veterinary wellbeing have been noted by other authors as a consequence of the pandemic (83).

The intersectional relationship of social class, race/ethnicity as well as sexual and gender identity with COVID 19 impacts is still being explored. Early analysis shows that minority populations have been disproportionately affected by the impacts of COVID-19. Racial minorities and lower income populations have an increased exposure to COVID-19, increased mortality, increased economic impacts, as well as decreased access to healthcare (84, 85). Gender inequalities were also exacerbated during the pandemic, as domestic violence increased during the pandemic, and women were subjected to greater economic impacts than their male counterparts (86). Sexual and gender minorities have also been shown to have increased mental health and wellbeing disturbances due to COVID-19 pandemic compared to straight and cis counterparts (87).

#### Role in practice

The respondents who identified their professional role as customer service or unlicensed veterinary assistant had higher percentages of serious psychological distress and considering suicide in the past 12 months than other roles in the profession. There was generally a trend that support staff and students had worse wellbeing and mental health than their DVM counterparts. This difference has been noted in other literature as well. In a 2021 survey, veterinary support staff were found to have higher suicidal ideation, suicide attempts, burnout, and serious psychological distress than their DVM counterparts (45). Another analysis found that while there was a significant general decrease in well-being across all professional categories, there was a greater impact in nursing staff and students, compared to their DVM counterparts (83). This is true across the healthcare workforce, as support workers are at higher risk for many negative health conditions (88).

In comparison to the role of associate vet, those who identified as a relief vet or “other” professional role were statistically significantly more likely to have suicidal thoughts. This difference has been noted in other literature as well. The Merck Animal Health Veterinary Wellbeing study found that relief vets had higher levels of psychological distress than other veterinarian roles (18).

### Limitations

To our knowledge, this is one of the first studies in veterinary medicine to identify multiple diverse social locations and their role in psychological distress, suicidal thinking, and suicidal behaviors during the COVID-19 pandemic. An important goal for the research team was to not “other” any demographic category. Because categories were not grouped together in a dichotomous “majority” and “other,” several statistical challenges existed. For example, due to small cell sizes in several race categories, there was not enough statistical power to make an accurate comparison between races. This challenge impacted seeing a clear picture of how race impacted psychological distress and suicide factors.

We intentionally recruited participants using “diversity” language in the recruitment advertisements (e.g., the title of the recruitment language was “Intersectionality and mental health in Vet Med”). This could have led to a self-selection bias to include only participants who either identified as diverse or who felt comfortable with intersectionality language. However, the self-report nature of this research may have resulted in respondents under, over, or incorrectly reporting their demographic or experiences of distress. This is entirely possible given issues of stigma related to our research questions. We recruited from multiple veterinary organizations (Table 1), some of which are designed to assist those seeking mental health support. This could have led to an oversampling of respondents experiencing serious psychological distress. Because of self-selection bias and small numbers in some demographic categories, it is important to interpret these findings with caution. Moreover, the findings should not be generalized to the entire population of individuals working in veterinary medicine.

### Implications of research findings

Attending to the demographic factors that may make an individual more susceptible to psychological distress and suicidal thinking is important to consider. Sexual identity and orientation, social class, being a support staff, and having a disability may make a person working in veterinary medicine more susceptible to experiencing distress. Cultural competence skills are important for veterinary practices to integrate into their training efforts toward creating veterinary communities of care and belonging. Additionally, we also found that those people who met criteria for psychological distress were 5 times more likely to endorse suicidal thinking. The K6 instrument is a very brief tool that has been widely used with many populations (41, 65, 89). It is free and open access (90). Our findings support other research that notes this tool can be helpful in identifying suicidal thinking (91, 92). Thus, the K6 may be a useful tool individuals can use to monitor their mental health. Paired with resources for seeking support and a culture that encourages accessing mental health care, this tool could be an important part of a suicide prevention approach in veterinary medicine.

## Data availability statement

The raw data supporting the conclusions of this article will be made available by the authors, without undue reservation.

## Ethics statement

The studies involving human participants were reviewed and approved by University of Tennessee Institutional Review Board. Written informed consent for participation was not required for this study in accordance with the national legislation and the institutional requirements.

## Author contributions

CJ, CT, KS, and ES created the project. KS created the survey and IRB application. CJ and CT recruited participants. KS, ES, AF, and WN did data analysis. All authors contributed to the article and approved the submitted version.

## Funding

The College of Veterinary Medicine at the University of Tennessee is providing the funds for open access publication fees.

## Conflict of interest

The authors declare that the research was conducted in the absence of any commercial or financial relationships that could be construed as a potential conflict of interest.

## Publisher’s note

All claims expressed in this article are solely those of the authors and do not necessarily represent those of their affiliated organizations, or those of the publisher, the editors and the reviewers. Any product that may be evaluated in this article, or claim that may be made by its manufacturer, is not guaranteed or endorsed by the publisher.

## References

[1] Blair A Hayes HM. Mortality patterns among US veterinarians, 1947–1977: an expanded study. Int J Epidemiol. (1982) 11:391–7. doi: 10.1093/ije/11.4.391, PMID: 7152791

[2] Miller D Staats S Partlo C. Discriminating positive and negative aspects of pet interaction: sex differences in the older population. Soc Indic Res. (1992) 27:363–74. doi: 10.1007/BF00303855

[3] Hem E Haldorsen T Aasland OG Tyssen R Vaglum P Ekeberg Ø. Suicide rates according to education with a particular focus on physicians in Norway 1960–2000. Psychol Med. (2005) 35:873–80. doi: 10.1017/S0033291704003344, PMID: 15997607

[4] Meltzer H Griffiths C Brock A Rooney C Jenkins R. Patterns of suicide by occupation in England and Wales: 2001–2005. Br J Psychiatry. (2008) 193:73–6. doi: 10.1192/bjp.bp.107.040550, PMID: 18700224

[5] Mammerickx M. Portrait of the contemporary Belgian veterinarian. “1. Documentary sources and evolution of Belgian veterinary medicine from its origins to the present,” in Annales De Medecine Veterinaire. Belgium: Annales Medecine Veterinaire. (1985). 441–6.

[6] Jones-Fairnie H Ferroni P Silburn S Lawrence D. Suicide in Australian veterinarians. Aust Vet J. (2008) 86:114–6. doi: 10.1111/j.1751-0813.2008.00277.x, PMID: 18363981

[7] Witte TK Spitzer EG Edwards N Fowler KA Nett RJ. Suicides and deaths of undetermined intent among veterinary professionals from 2003 through 2014. J Am Vet Med Assoc. (2019) 255:595–608. doi: 10.2460/javma.255.5.595, PMID: 31429646 PMC6933287

[8] Nett RJ Witte TK Holzbauer SM Elchos BL Campagnolo ER Musgrave KJ. Notes from the field: prevalence of risk factors for suicide among veterinarians—United States, 2014. MMWR Morb Mortal Wkly Rep. (2015) 64:131–2. PMID: 25674997 PMC4584691

[9] Pratt LA Dey AN Cohen AJ. Characteristics of adults with serious psychological distress as measured by the K6 scale: United States, 2001-04. Adv Data. (2007) 382:1–18. 17432488

[10] Ouedraogo FB Lefebvre SL Hansen CR Brorsen BW. Compassion satisfaction, burnout, and secondary traumatic stress among full-time veterinarians in the United States (2016–2018). J Am Vet Med Assoc. (2021) 258:1259–70. doi: 10.2460/javma.258.11.1259, PMID: 33978434

[11] Vande Griek OH Clark MA Witte TK Nett RJ Moeller AN Stabler ME. Development of a taxonomy of practice-related stressors experienced by veterinarians in the United States. J Am Vet Med Assoc. (2018) 252:227–33. doi: 10.2460/javma.252.2.227, PMID: 29319445 PMC5985521

[12] Volk JO Schimmack U Strand EB Vasconcelos J Siren CW. Executive summary of the Merck Animal Health Veterinarian Wellbeing Study II. J Am Vet Med Assoc. (2020) 256:1237–44. doi: 10.2460/javma.256.11.1237, PMID: 32412878

[13] Bartram DJ Baldwin DS. Veterinary surgeons and suicide: a structured review of possible influences on increased risk. Vet Rec. (2010) 166:388–97. doi: 10.1136/vr.b4794, PMID: 20348468

[14] Witte TK Correia CJ Angarano D. Experience with euthanasia is associated with fearlessness about death in veterinary students. Suicide Life Threat Behav. (2013) 43:125–38. doi: 10.1111/sltb.12000, PMID: 23278546

[15] Oshio A Taku K Hirano M Saeed G. Resilience and big five personality traits: a meta-analysis. Pers Individ Differ. (2018) 127:54–60. doi: 10.1016/j.paid.2018.01.048

[16] Everaerd D Klumpers F van Wingen G Tendolkar I Fernández G. Association between neuroticism and amygdala responsivity emerges under stressful conditions. NeuroImage. (2015) 112:218–24. doi: 10.1016/j.neuroimage.2015.03.014, PMID: 25776217

[17] Vidal-Arenas V Bravo AJ Ortet-Walker J Ortet G Mezquita L Ibáñez MI. Neuroticism, rumination, depression and suicidal ideation: a moderated serial mediation model across four countries. Int J Clin Health Psychol. (2022) 22:100325. doi: 10.1016/j.ijchp.2022.100325 35950010 PMC9343412

[18] Volk JO Schimmack U Strand EB Lord LK Siren CW. Executive summary of the Merck Animal Health Veterinary Wellbeing Study. J Am Vet Med Assoc. (2018) 252:1231–8. doi: 10.2460/javma.252.10.1231, PMID: 29701527

[19] Holden CL. Characteristics of veterinary students: perfectionism, personality factors, and resilience. J Vet Med Educ. (2020) 47:488–96. doi: 10.3138/jvme.0918-111r, PMID: 32412364

[20] Crane MF Phillips JK Karin E. Trait perfectionism strengthens the negative effects of moral stressors occurring in veterinary practice. Aust Vet J. (2015) 93:354–60. doi: 10.1111/avj.12366, PMID: 26412116

[21] Hughes JL Camden AA Yangchen T. Rethinking and updating demographic questions: guidance to improve descriptions of research samples. Psi Chi J Psychol Res. (2016) 21:138–51. doi: 10.24839/2164-8204.JN21.3.138

[22] Tomasi SE Fechter-Leggett ED Edwards NT Reddish AD Crosby AE Nett RJ. Suicide among veterinarians in the United States from 1979 through 2015. J Am Vet Med Assoc. (2019) 254:104–12. doi: 10.2460/javma.254.1.104, PMID: 30668293 PMC6417412

[23] Nett RJ Witte TK Holzbauer SM Elchos BL Campagnolo ER Musgrave KJ. Risk factors for suicide, attitudes toward mental illness, and practice-related stressors among US veterinarians. J Am Vet Med Assoc. (2015) 247:945–55. doi: 10.2460/javma.247.8.945, PMID: 26421408

[24] Witte TK Kramper S Carmichael KP Chaddock M Gorczyca K. A survey of negative mental health outcomes, workplace and school climate, and identity disclosure for lesbian, gay, bisexual, transgender, queer, questioning, and asexual veterinary professionals and students in the United States and United Kingdom. J Am Vet Med Assoc. (2020) 257:417–31. doi: 10.2460/javma.257.4.417, PMID: 32715886

[25] Krieger N Kosheleva A Waterman PD Chen JT Koenen K. Racial discrimination, psychological distress, and self-rated health among US-born and foreign-born Black Americans. Am J Public Health. (2011) 101:1704–13. doi: 10.2105/AJPH.2011.300168, PMID: 21778504 PMC3154215

[26] Asdigian NL Bear UR Beals J Manson SM Kaufman CE. Mental health burden in a national sample of American Indian and Alaska native adults: differences between multiple-race and single-race subgroups. Soc Psychiatry Psychiatr Epidemiol. (2018) 53:521–30. doi: 10.1007/s00127-018-1494-1, PMID: 29470596

[27] Williams DR Lawrence JA Davis BA. Racism and health: evidence and needed research. Annu Rev Public Health. (2019) 40:105–25. doi: 10.1146/annurev-publhealth-040218-043750, PMID: 30601726 PMC6532402

[28] Veterinarians and Veterinary Technologists. Datausa. Available at: https://datausa.io/profile/soc/veterinarians?compare=veterinary-technologists-and-technicians (Accessed November 21, 2022).

[29] Annual data report. Available at: https://www.aavmc.org/wp-content/uploads/2022/08/2022-AAVMC-Annual-Data-Report-8.8Update.pdf

[30] FACTS. AAMC. Available at: https://www.aamc.org/data-reports/students-residents/interactive-data/2022-facts-enrollment-graduates-and-md-phd-data (Accessed November 21, 2022).

[31] Williams MR Do DP. The compounded burden of poverty on mental health for people with disabilities. Soc Work Public Health. (2021) 36:419–31. doi: 10.1080/19371918.2021.1905579, PMID: 33832403

[32] Andrews EE. Disability as diversity: developing cultural competence. USA: Oxford University Press (2019).

[33] Adams M Brown S. Towards inclusive learning in higher education: developing curricula for disabled students. London: Routledge (2006).

[34] Lindström M Rosvall M. Marital status, social capital, economic stress, and mental health: a population-based study. Soc Sci J. (2012) 49:339–42. doi: 10.1016/j.soscij.2012.03.004 22925881

[35] Simon RW. Revisiting the relationships among gender, marital status, and mental health. AJS. (2002) 107:1065–96. doi: 10.1086/339225, PMID: 12227382

[36] Phillips-Miller DL Campbell NJO Morrison CR. Work and family: satisfaction, stress, and spousal support. J Employ Couns. (2000) 37:16–30. doi: 10.1002/j.2161-1920.2000.tb01023.x, PMID: 36006738

[37] Chigerwe M Barter L Dechant JE Dear JD Boudreaux KA. A preliminary study on assessment of wellbeing among veterinary medical house officers. PLoS One. (2021) 16:e0253111. doi: 10.1371/journal.pone.0253111, PMID: 34166405 PMC8224950

[38] Panchal N Kamal R Cox C Garfield R Orgera K. The implications of COVID-19 for mental health and substance use Kaiser Family Foundation (2020). 21 p Available at: https://pameladwilson.com/wp-content/uploads/4_5-2021-The-Implications-of-COVID-19-for-Mental-Health-and-Substance-Use-_-KFF-1.pdf.

[39] CDC. Interim infection prevention and control guidance for veterinary clinics treating companion animals during the COVID-19 response. Centers for Disease Control (2020). Available at: https://pesquisa.bvsalud.org/global-literature-on-novel-coronavirus-2019-ncov/resource/pt/grc-739910 (Accessed December 9, 2021).

[40] Qualtrics. Qualtrics. (2021). Available at: https://www.qualtrics.com

[41] Kessler RC Barker PR Colpe LJ Epstein JF Gfroerer JC Hiripi E. Screening for serious mental illness in the general population. Arch Gen Psychiatry. (2003) 60:184–9. doi: 10.1001/archpsyc.60.2.184, PMID: 12578436

[42] Brennan RL. Generalizability theory. Educational Measurement: Issues and Practice. (1992) 11:27–34.

[43] Haney JL. Suicidality risk among adult sexual minorities: results from a cross-sectional population-based survey. J Gay Lesbian Soc Serv. (2021) 33:250–71. doi: 10.1080/10538720.2021.1875946, PMID: 17907860

[44] DiStefano C Shi D Morgan GB. Collapsing categories is often more advantageous than modeling sparse data: investigations in the CFA framework. Struct Equ Modeling. (2021) 28:237–49. doi: 10.1080/10705511.2020.1803073

[45] Volk JO Schimmack U Strand EB Reinhard A Vasconcelos J Hahn J. Executive summary of the Merck Animal Health Veterinarian Wellbeing Study III and Veterinary Support Staff Study. J Am Vet Med Assoc. (2022) 260:1–7. doi: 10.2460/javma.22.03.0134, PMID: 35943942

[46] Dubé JP Smith MM Sherry SB Hewitt PL Stewart SH. Suicide behaviors during the COVID-19 pandemic: a meta-analysis of 54 studies. Psychiatry Res. (2021) 301:113998. doi: 10.1016/j.psychres.2021.113998, PMID: 34022657 PMC9225823

[47] Czeisler MÉ Lane RI Wiley JF Czeisler CA Howard ME Rajaratnam SMW. Follow-up survey of US adult reports of mental health, substance use, and suicidal ideation during the COVID-19 pandemic, September 2020. JAMA Netw Open. (2021) 4:e2037665. doi: 10.1001/jamanetworkopen.2020.37665, PMID: 33606030 PMC7896196

[48] Buspavanich P Lech S Lermer E Fischer M Berger M Vilsmaier T. Well-being during COVID-19 pandemic: a comparison of individuals with minoritized sexual and gender identities and cis-heterosexual individuals. PLoS One. (2021) 16:e0252356. doi: 10.1371/journal.pone.0252356, PMID: 34101746 PMC8186787

[49] Guz S Kattari SK Atteberry-Ash B Klemmer CL Call J Kattari L. Depression and suicide risk at the cross-section of sexual orientation and gender identity for youth. J Adolesc Health. (2021) 68:317–23. doi: 10.1016/j.jadohealth.2020.06.008, PMID: 32680801

[50] Lipson SK Raifman J Abelson S Reisner SL. Gender minority mental health in the U.S.: results of a national survey on college campuses. Am J Prev Med. (2019) 57:293–301. doi: 10.1016/j.amepre.2019.04.025, PMID: 31427032

[51] Barboza GE Dominguez S Chance E. Physical victimization, gender identity and suicide risk among transgender men and women. Prev Med Rep. (2016) 4:385–90. doi: 10.1016/j.pmedr.2016.08.003, PMID: 27547721 PMC4986045

[52] Cogan CM Scholl JA Cole HE Davis JL. The moderating role of community resiliency on suicide risk in the transgender population. J LGBT Issues Couns. (2020) 14:2–17. doi: 10.1080/15538605.2020.1711291, PMID: 35465758

[53] Kosciw JG Palmer NA Kull RM. Reflecting resiliency: openness about sexual orientation and/or gender identity and its relationship to well-being and educational outcomes for LGBT students. Am J Community Psychol. (2015) 55:167–78. doi: 10.1007/s10464-014-9642-6, PMID: 24691967

[54] Crawford TN Ridner SL. Differences in well-being between sexual minority and heterosexual college students. J LGBT Youth. (2018) 15:243–55. doi: 10.1080/19361653.2018.1470954, PMID: 37452819

[55] Perales F. The costs of being “different”: sexual identity and subjective wellbeing over the life course. Soc Indic Res. (2016) 127:827–49. doi: 10.1007/s11205-015-0974-x

[56] Swannell S Martin G Page A. Suicidal ideation, suicide attempts and non-suicidal self-injury among lesbian, gay, bisexual and heterosexual adults: findings from an Australian national study. Aust N Z J Psychiatry. (2016) 50:145–53. doi: 10.1177/0004867415615949, PMID: 26631718

[57] Ross LE Salway T Tarasoff LA JM MK Hawkins BW Fehr CP. Prevalence of depression and anxiety among bisexual people compared to gay, lesbian, and heterosexual individuals: a systematic review and meta-analysis. J Sex Res. (2018) 55:435–56. doi: 10.1080/00224499.2017.1387755 29099625

[58] Hottes TS Bogaert L Rhodes AE Brennan DJ Gesink D. Lifetime prevalence of suicide attempts among sexual minority adults by study sampling strategies: a systematic review and meta-analysis. Am J Public Health. (2016) 106:e1–e12. doi: 10.2105/AJPH.2016.303088, PMID: 27049424 PMC4985071

[59] Salway T Ross LE Fehr CP Burley J Asadi S Hawkins B. A systematic review and meta-analysis of disparities in the prevalence of suicide ideation and attempt among bisexual populations. Arch Sex Behav. (2019) 48:89–111. doi: 10.1007/s10508-018-1150-6, PMID: 29492768

[60] Chan RCH Operario D Mak WWS. Bisexual individuals are at greater risk of poor mental health than lesbians and gay men: the mediating role of sexual identity stress at multiple levels. J Affect Disord. (2020) 260:292–301. doi: 10.1016/j.jad.2019.09.020, PMID: 31521866

[61] McInroy LB Beaujolais B Leung VWY Craig SL Eaton AD Austin A. Comparing asexual and non-asexual sexual minority adolescents and young adults: stressors, suicidality and mental and behavioural health risk outcomes. Psychol Sex. (2022) 13:387–403. doi: 10.1080/19419899.2020.1806103

[62] Borgogna NC McDermott RC Aita SL Kridel MM. Anxiety and depression across gender and sexual minorities: implications for transgender, gender nonconforming, pansexual, demisexual, asexual, queer, and questioning individuals. Psychol Sex Orientat Gend Divers. (2019) 6:54–63. doi: 10.1037/sgd0000306

[63] Viertiö S Kiviruusu O Piirtola M Kaprio J Korhonen T Marttunen M. Factors contributing to psychological distress in the working population, with a special reference to gender difference. BMC Public Health. (2021) 21:611. doi: 10.1186/s12889-021-10560-y 33781240 PMC8006634

[64] Kivimäki M Batty GD Pentti J Shipley MJ Sipilä PN Nyberg ST. Association between socioeconomic status and the development of mental and physical health conditions in adulthood: a multi-cohort study. Lancet Public Health. (2020) 5:e140–9. doi: 10.1016/S2468-2667(19)30248-8, PMID: 32007134

[65] Gullett LR Alhasan DM Jackson WB 2nd Jackson CL. Employment industry and occupational class in relation to serious psychological distress in the United States. Int J Environ Res Public Health. (2022) 19:8376. doi: 10.3390/ijerph19148376, PMID: 35886224 PMC9320061

[66] Eisenberg-Guyot J Hajat A. Under capital’s thumb: longitudinal associations between relational social class and health. J Epidemiol Community Health. (2020) 74:453–9. doi: 10.1136/jech-2019-213440, PMID: 32086371 PMC7173644

[67] Gerada C. Doctors, suicide and mental illness. BJPsych Bull. (2018) 42:165–8. doi: 10.1192/bjb.2018.11, PMID: 29712575 PMC6436060

[68] Rendall MS Weden MM Favreault MM Waldron H. The protective effect of marriage for survival: a review and update. Demography. (2011) 48:481–506. doi: 10.1007/s13524-011-0032-5, PMID: 21526396

[69] Westrick-Payne K Manning W. Marriage, divorce, and the COVID-19 pandemic in the U.S. KY: Bowling Green State University (2022).

[70] Pietromonaco PR Overall NC. Applying relationship science to evaluate how the COVID-19 pandemic may impact couples’ relationships. Am Psychol. (2021) 76:438–50. doi: 10.1037/amp0000714, PMID: 32700937

[71] Strine TW Dhingra SS Okoro CA Zack MM Balluz LS Berry JT. Serious psychological distress among adults with and without disabilities. Int J Public Health. (2009) 54:9–15. doi: 10.1007/s00038-009-0001-6 19363587

[72] Tynan A. Veterinarians with disabilities: an international issue. J Vet Med Educ. (2004) 31:22–7. doi: 10.3138/jvme.31.1.22, PMID: 15962245

[73] Odunayo A Ng ZY. Valuing diversity in the team. Vet Clin North Am Small Anim Pract. (2021) 51:1009–40. doi: 10.1016/j.cvsm.2021.05.002, PMID: 34334163

[74] Timmenga FSL Jansen W Turner PV De Briyne N. Mental well-being and diversity, equity, and inclusiveness in the veterinary profession: pathways to a more resilient profession. Front Vet Sci. (2022) 9:888189. doi: 10.3389/fvets.2022.888189, PMID: 35967992 PMC9372717

[75] Reijula K Räsänen K Hämäläinen M Juntunen K Lindbohm ML Taskinen H. Work environment and occupational health of Finnish veterinarians. Am J Ind Med. (2003) 44:46–57. doi: 10.1002/ajim.10228, PMID: 12822135

[76] Fritschi L Morrison D Shirangi A Day L. Psychological well-being of Australian veterinarians. Aust Vet J. (2009) 87:76–81. doi: 10.1111/j.1751-0813.2009.00391.x, PMID: 19245615

[77] Dow MQ Chur-Hansen A Hamood W Edwards S. Impact of dealing with bereaved clients on the psychological wellbeing of veterinarians. Aust Vet J. (2019) 97:382–9. doi: 10.1111/avj.12842, PMID: 31364771

[78] Thomas ML Kaufmann CN Palmer BW Depp CA Martin AS Glorioso DK. Paradoxical trend for improvement in mental health with aging: a community-based study of 1,546 adults aged 21–100 years. J Clin Psychiatry. (2016) 77:e1019–25. doi: 10.4088/JCP.16m10671 27561149 PMC5461877

[79] Thomas-Hawkins C Zha P Flynn L Ando S. Effects of race, workplace racism, and COVID worry on the emotional well-being of hospital-based nurses: a dual pandemic. Behav Med. (2022) 48:95–108. doi: 10.1080/08964289.2021.1977605, PMID: 35318891

[80] Snyder CR Schwartz MR. Experiences of workplace racial discrimination among people of color in healthcare professions. J Cult Divers. (2019) 26. Available at: https://search.ebscohost.com/login.aspx?direct=true&profile=ehost&scope= site&authtype=crawler&jrnl=10715568&AN=139005697&h=7MJtI2s%2B64w8esXQMILiR69H rhXJEqgoNMXiuFV4vVD0edtOMIJUdzFh6JEBm6jUHDQjIkIcD9Q ERNtE8mjJWA%3D%3D&crl=c

[81] Zacher H Rudolph CW. Individual differences and changes in subjective wellbeing during the early stages of the COVID-19 pandemic. Am Psychol. (2021) 76:50–62. doi: 10.1037/amp0000702, PMID: 32700938

[82] Quain A Mullan S McGreevy PD Ward MP. Frequency, stressfulness and type of ethically challenging situations encountered by veterinary team members during the COVID-19 pandemic. Front Vet Sci. (2021) 8:647108. doi: 10.3389/fvets.2021.752388, PMID: 33912607 PMC8071942

[83] Mair TS Mountford DR Radley R Lockett E Parkin TD. Mental wellbeing of equine veterinary surgeons, veterinary nurses and veterinary students during the COVID-19 pandemic. Equine Vet Educ. (2021) 33:15–23. doi: 10.1111/eve.13399

[84] Ruprecht MM Wang X Johnson AK Xu J Felt D Ihenacho S. Evidence of social and structural COVID-19 disparities by sexual orientation, gender identity, and race/ethnicity in an urban environment. J Urban Health. (2021) 98:27–40. doi: 10.1007/s11524-020-00497-9, PMID: 33259027 PMC7706696

[85] Lopez MH Rainie L Budiman A. Financial and health impacts of COVID-19 vary widely by race and ethnicity. (2020). Available at: https://policycommons.net/artifacts/616219/financial-and-health-impacts-of-covid-19-vary-widely-by-race-and-ethnicity/1596847/ (Accessed December 23, 2022).

[86] Piquero AR Jennings WG Jemison E Kaukinen C Knaul FM. Domestic violence during the COVID-19 pandemic—evidence from a systematic review and meta-analysis. J Crim Justice. (2021) 74:101806. doi: 10.1016/j.jcrimjus.2021.101806, PMID: 36281275 PMC9582712

[87] Peterson ZD Vaughan EL Carver DN. Sexual identity and psychological reactions to COVID-19. Traumatology. (2021) 27:6–13. doi: 10.1037/trm0000283, PMID: 34816161

[88] Silver SR Li J Marsh SM Carbone EG. Prepandemic mental health and well-being: differences within the health care workforce and the need for targeted resources. J Occup Environ Med. (2022) 64:1025–35. doi: 10.1097/JOM.0000000000002630, PMID: 36472564 PMC9722331

[89] Tomitaka S Kawasaki Y Ide K Akutagawa M Yamada H Ono Y. Characteristic distribution of the total and individual item scores on the Kessler screening scale for psychological distress (K6) in US adults. BMC Psychiatry. (2017) 17:290. doi: 10.1186/s12888-017-1449-1 28784101 PMC5545851

[90] National comorbidity survey. Available at: https://www.hcp.med.harvard.edu/ncs/k6_scales.php (Accessed March 26, 2023).

[91] Meyer D Abbott JA Rehm I Bhar S Barak A Deng G. Development of a suicidal ideation detection tool for primary healthcare settings: using open access online psychosocial data. Telemed J E Health. (2017) 23:273–81. doi: 10.1089/tmj.2016.0110, PMID: 27662524

[92] Naito Y Enomoto N Kameno Y Yamasue H Suda T Hotta Y. Kessler psychological distress (K6) questionnaire scores can predict autistic traits and the current and prospective suicidal ideation in medical university students: a prospective study. SAGE Open. (2021) 11:2158244021994590. doi: 10.1177/2158244021994590

