# Supplementary material for: Relationships between diversity demographics, psychological distress, and suicidal thinking in the veterinary profession: a nationwide cross-sectional study during COVID-19
Source: Front Vet Sci. 2023 Aug 16;10:1130826. doi: 10.3389/fvets.2023.1130826 (PMC10469311; doi:10.3389/fvets.2023.1130826)
Supplement: Supplementary file 1 [file Data_Sheet_1.docx]

**Appendix**

Demographic questions asked in diversity/psychological distress study:

(Race) What race do you identify with?

· African or Descendant of the African Diaspora (Black, African-American, Afro-Caribbean, African, Afro-Latinx, Middle Eastern and Northern African, etc.) (12)

· Native or Indigenous (Alaska Native, American Indian, Native Hawaiian etc.) (13)

· Asian (Southeast, South, East, etc.) (14)

· Pacific Islanders (Polynesian, Melanesian, Micronesian) (15)

· Middle Eastern and Northern African (Algerian, Egyptian, Iranian, Moroccan, Palestinian, Saudi Arabian, Israeli, etc.) (16)

· White (17)

· Two or more races (18)

· Prefer not to answer (19)

· Not listed (20)

(Ethnicity) What ethnicity do you identify with?

· Hispanic (Argentinian, Bolivian, Mexican, Spanish, Columbian, Cuban, Dominican, Ecuadorian, Puerto Rican, etc.) (4)

· Other Hispanic, Latinx, or Spanish Origin (5)

· Non-Hispanic (6)

· Prefer not to answer (7)

(Gender) How do you currently describe your gender?

· Cisgender Man or Male or Masculine (1)

· Transgender Man or Male or Masculine (2)

· Transgender Woman or Female or Feminine (3)

· Cisgender Woman or Female or Feminine (4)

· Gender non-conforming or Gender queer (5)

· Gender fluid (6)

· Intersex or other related terms (7)

· Agendered (8)

· Questioning (9)

· (Gendertxt) Other (10) ________________________________________________

· Prefer not to answer (11)

(SexOrient) Do you consider yourself to be:

· Heterosexual or straight (1)

· Homosexual: Gay or lesbian (2)

· BI+/Non-monosexual: Such as Bisexual, Pansexual, Omnisexual (3)

· Fluid (4)

· Queer (5)

· Questioning (6)

· Asexual (7)

· (SexOrienttxt) Other (8) ________________________________________________

· Prefer not to answer (9)

(Age) What is your age in years?

________________________________________________________________

(Marital) What is your current marital status?

· Legally married or in a committed relationship (1)

· Separated (2)

· Divorced (3)

· Widowed (4)

· Never married (5)

(Children) How many children do you have? (Include biological, adopted and step-children)

· 0 (1)

· 1 (2)

· 2 (3)

· 3 (4)

· 4 or more (5)

(Disability) Are you currently experiencing a disability, impairment or significant disease?

· No (1)

· Yes (2)

· Prefer not to answer (3)

Q43 If yes, which of the following apply?

· (Disablesense) Sensory impairment (vision or hearing) (1)

· (Disablemobile) Mobility impairment (2)

· (Disablelearn) Learning disability (3)

· (Disablemental) Mental health disorder (4)

· (Disablephysical) Physical illness (cancer, diabetes, etc) (5)

· (Disabilityothr) Other (6)

(Disabilitytxt) ________________________________________________

(Socialclass) Which social class do you identify with?

· Poor (1)

· Working class (2)

· Middle class (3)

· Upper middle class (4)

· Affluent (5)

· Prefer not to answer (6)

(Annual) What is your annual household income?

· $25,000 or less (1)

· $25,001-$50,000 (2)

· $50,001-$75,000 (3)

· $75,001-$100,000 (4)

· $100,001-$150,000 (5)

· $150,001-$200,000 (6)

· $200,001 or more (7)

· Prefer not to answer (8)

(Role) What is your primary professional role?

· Customer service/Reception (1)

· Unlicensed Veterinary Assistant/Kennel (2)

· Credentialed Technician (3)

· Management (4)

· Associate Veterinarian (5)

· Relief Veterinarian (6)

· Practice Owner (7)

· Intern/Resident (8)

· Student (9)

· (Roletxt) Other (10) ________________________________________________
